# CRISPR-mediated RNA base editing: a promising strategy to rescue deafness

**DOI:** 10.1038/s41392-023-01349-z

**Published:** 2023-02-27

**Authors:** Wenyi Liu, Min Wu, Guanwang Shen

**Affiliations:** 1 Wound Trauma Medical Center, State Key Laboratory of Trauma, Burns and Combined Injury, Daping Hospital, Army Medical University, Chongqing, 400042 China; 2 Wenzhou Institute, University of Chinese Academy of Sciences, Wenzhou, Zhejiang 325000 China; 3 Integrative Science Center of Germplasm Creation in Western China (CHONGQING) Science City & Southwest University, Biological Science Research Center, Southwest University, Chongqing, 400716 China

**Keywords:** Gene therapy, Medical genetics

**Fig. 1:**
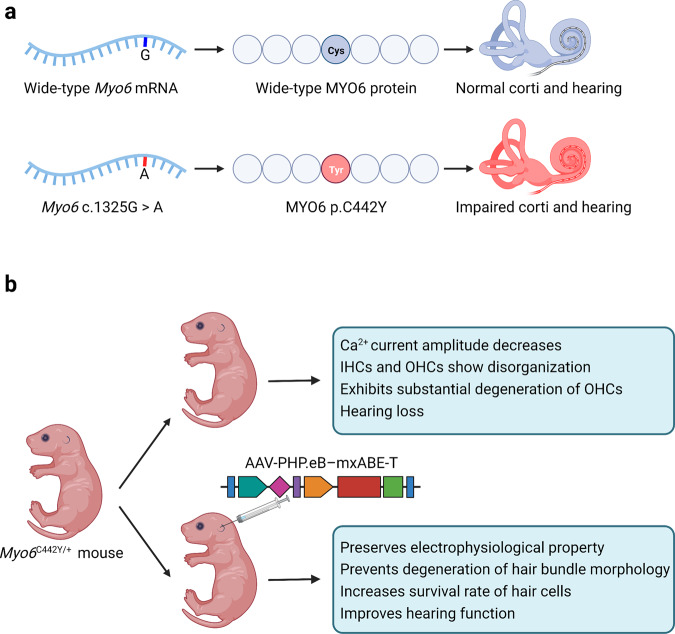
Programmable base editing rescues auditory function of an autosomal dominant hearing loss disease. **a** A single G to A mutation at 1325 locus in the *MYO6* gene causes corti and hearing impairment. **b** AAV-PHP.eB-mxABE-T treatment in *Myo6*^*C442Y/+*^ mouse model. Xiao et al. co-packaged mxABE and gRNA into AAV-PHP.eB to correct the mutation in *Myo6*^*C442Y/+*^ mice resulting in improved hearing function. This figure was created with BioRender.com

A recent study published in Science Translational Medicine by Xiao et al. reported the use of mini dCas13X-based adenosine base editor (mxABE) to correct a mutant transcript and rescued the auditory function of an autosomal dominant hearing loss disease (Fig. 1).^1^

Many mutations of human *MYO6* gene lead to sensorineural hearing loss (SNHL) with no effective treatment. As a semi-dominant inheritance mouse model, the heterozygous *Myo6*^*C442Y/+*^ mice recapitulated the progressive SNHL in human.^2^ Xiao et al. used RNA base editing system, basically composed of base editors and guide RNA (gRNA) to treat this mouse model. To screen optimal RNA base editors and gRNAs for *Myo6*^*C442Y*^ correction, Xiao et al. overexpressed *Myo6*^*C442Y*^ RNA in the human embryonic kidney (HEK) 293T cells, cotransfected with different RNA base editors (mini dCas13X or Cas13b was fused with ADAR2dd v1 or ADAR2dd v2) and gRNAs targeting C442Y mutation. The mini dCas13X fused with ADARdd-v1(mxABE-v1) showed high editing efficiency with low off-target.

Next, Xiao et al. packaged mxABE-v1 and gRNA targeting C442Y mutation into adeno-associated virus (AAV)-PHP.eB (hereafter referred to as AAV-mxABE-T) and injected it into the inner ear of *Myo6*^*C442Y/+*^ mice at postnatal day 0 (P0) to P2. Significant base editing can be detected 2 weeks after the injection, and the AAV-mxABE-T-treatment resulted in the preservation of electrophysiological property, embodied by partial recovery of decreased Ca^2+^ current amplitude in inner hair cells (IHCs). Importantly, compared to Myo6^*C442Y/+*^ mice with substantial degeneration of outer hair cells (OHCs) and substantial disorganization of surviving IHCs and OHCs, the *Myo6*^*C442Y/+*^ mice treated with AAV-mxABE-T showed more OHCs in the middle and basal turns of the cochlea, and more organized hair bundles in OHCs and IHCs. Furthermore, AAV-mxABE-T treatment led to significantly decreased ABR and DPOAE thresholds, indicating an improvement in hearing function. Together, AAV-mxABE-T treatment attenuates some typical microscopic lesions of *Myo6*^*C442Y/+*^ mice in vivo, which may rescue the auditory function of the diseased mice.

SNHL is a common disease with no effective treatment. However, the use of drugs, stem cells and gene therapy is struggling to develop as potential strategies for improving hearing cells or auditory function in SNHL patients. Since genetic factors contribute to approximately half of the congenital SNHL cases, gene therapy is potentially important means to inhibit the disease progression and improve the symptom. CRISPR-Cas systems are the most widely used gene editing tools, and base editing is a newer application of these systems to generate precise point mutations in DNA or RNA. The permanent genomic alterations and requirement of a protospacer adjacent motif (PAM) at the editing site may limit the use of DNA base editors, such as Cas9 and Cas12. Since RNA editing is reversible and non-heritable, CRISPR-Cas13 system, specifically targets and edits RNA, should better meet the requirements of ethics, especially in clinical application.^3^ Although Cas13a to c have shown varying dependence on a protospacer flanking sequence (PFS) for efficient RNA targeting, Cas13d and Cas13X have no PFS bias. Coupled with its compact size, the mini base editor mxABE provides possibility to treat genetic diseases in vivo via a single AAV vector. By packing mxABE-v1 and gRNA into an AAV, Xiao et al. directly corrected *Myo6*^*C442Y/+*^ mutation in vivo without breaking double-strand DNA and bringing in eternal genomic changes.

The delivery method is one of the most important issues with the use of mxABE. AAV is considered a promising virus to deliver gene therapy vectors in vivo, but the results of three children’s death in a clinical trial for X-linked myotubular myopathy caused great worry about the safety of recombinant AAVs.^4^ Since RNA editing is temporary and reversible, repeated AAV injection may be needed in some process of treatment, an optimal highly infectious serotype, an appropriate time, dosage, and method to transfer the virus need to be selected. Besides, large preclinical data about effective methods of controlling the AAVs immunogenicity are required before clinical translation, and methods to reduce the anti-AAV humoral response are required, such as inhibiting B cell activation, modifying AAV surface, and transiently reducing IgG levels. In addition, alternative strategies for AAV methods are being exploited. Several studies have shown that Lipid nanoparticles (LNPs) play an important role in mRNA vaccines against COVID-19 and other nucleic acid-based therapies, which hold promises for their potential to deliver molecules of mxABE and gRNA to the target cells. Moreover, to cure the patients suffering from *Myo6*,^*C442Y/+*^ extended preclinical and clinical studies are required to identify most suitable delivery methods with high efficiency and specificity, and low safety risks.

Because of the off-target effects, strategies for improving the efficiency and fidelity of mxABE are required. An appropriate gRNA is important, even the best-designed gRNA sometimes needs engineering by chemical modifications or other methods to target more specifically. On the other hand, Cas endonucleases are equally important and have been developed rapidly. Tong et al. reported a high-fidelity Cas13X variant exhibiting efficient on-target activity but markedly reduced collateral activity.^5^ We expect this variant and other high-fidelity Cas endonucleases to be used to optimize the therapy strategy. Furthermore, it is our opinion that for the foreseeable future, many new anti-CRISPR proteins or small molecule drugs will be found and improved. Either of them, acts as a failsafe or an adjuvant to reduce off-target effects in patients, will make it possible to block the activities of mini dCas13X when it causes side-effects or after required gene editing has achieved to result in precise RNA editing.

Lastly, widely recognized international laws and regulations on acceptable therapeutic interventions in humans are required, and tougher measures should be introduced to avoid ethical issues for the clinical application of CRISPR-Cas system.

Taken together, this result from Xiao et al. demonstrates that mxABE has the ability to correct the *Myo6*^*C442Y*^ mutation and partially repair phenotypic traits in a mouse model. It provides a potential utility of RNA editing tools as a possible therapeutic approach to cure sensorineural hearing loss and other genetic diseases.
